# The Role of Artificial Intelligence in Prospective Real-Time Histological Prediction of Colorectal Lesions during Colonoscopy: A Systematic Review and Meta-Analysis

**DOI:** 10.3390/diagnostics13203267

**Published:** 2023-10-20

**Authors:** Bhamini Vadhwana, Munir Tarazi, Vanash Patel

**Affiliations:** 1 Department of Surgery and Cancer, Imperial College London, Du Cane Road, London W12 0HS, UK; 2 West Hertfordshire Hospital NHS Trust, Vicarage Road, Watford WD18 0HB, UK

**Keywords:** artificial intelligence, machine learning, colonoscopy, colorectal cancer, colorectal polyps

## Abstract

Artificial intelligence (AI) presents a novel platform for improving disease diagnosis. However, the clinical utility of AI remains limited to discovery studies, with poor translation to clinical practice. Current data suggests that 26% of diminutive pre-malignant lesions and 3.5% of colorectal cancers are missed during colonoscopies. The primary aim of this study was to explore the role of artificial intelligence in real-time histological prediction of colorectal lesions during colonoscopy. A systematic search using MeSH headings relating to “AI”, “machine learning”, “computer-aided”, “colonoscopy”, and “colon/rectum/colorectal” identified 2290 studies. Thirteen studies reporting real-time analysis were included. A total of 2958 patients with 5908 colorectal lesions were included. A meta-analysis of six studies reporting sensitivities (95% CI) demonstrated that endoscopist diagnosis was superior to a computer-assisted detection platform, although no statistical significance was reached (*p* = 0.43). AI applications have shown encouraging results in differentiating neoplastic and non-neoplastic lesions using narrow-band imaging, white light imaging, and blue light imaging. Other modalities include autofluorescence imaging and elastic scattering microscopy. The current literature demonstrates that despite the promise of new endoscopic AI models, they remain inferior to expert endoscopist diagnosis. There is a need to focus developments on real-time histological predictions prior to clinical translation to demonstrate improved diagnostic capabilities and time efficiency.

## 1. Introduction

Artificial intelligence (AI) presents a novel platform for improving disease diagnosis and improve clinician performance. However, the clinical utility of AI remains limited to discovery studies, with poor translation to clinical practice. AI encompasses machine learning and deep learning methods. Machine learning methods allow the system to be trained in characterising key features, differentiating samples, and subsequently exploiting this to classify new information [1,2]. Support vector machines require manual input of target features to train the system to identify and discriminate features for analysis [3]. Convolutional neural networks (CNNs) are supervised machine learning algorithms that function from multiple input features, which are collated to produce a final classification output [3]. Computer-aided diagnosis (CAD) with AI systems has been investigated in gastrointestinal endoscopy. The vision for the integration of AI in endoscopic procedures is the improvement of diagnostic accuracy, time efficiency, and facilitating decision-making for polyp resection; this remains in the exploratory phase; however, CAD has been heavily researched in radiological imaging, such as colorectal cancer lymph node metastases, lymphovascular invasion, and their associated survival and prognostic outcomes.

In rectal cancer, AI applications in pelvic magnetic resonance imaging (MRI) have shown promise in accurate staging of cancer, lymph node detection, and predictions of response to neoadjuvant chemoradiotherapy [4]. Another advanced area in AI is breast cancer imaging. The Wisconsin Breast Cancer Dataset allows the training of AI models for early breast cancer detection. Novel platforms, such as the least-squares support vector machine, with a 98.5% classification accuracy, are being used in national screening programmes [5]. Other AI-based technologies are developing in prostate cancer for treatment response prediction, lung cancer for early detection to improve survival outcomes, and other fields, such as classifying genetic abnormalities from genetic data [6,7,8].

Endoscopy is a complex procedure that has become widely used for diagnostics, including screening and surveillance of gastrointestinal pathology. More recently, its role in therapeutic procedures in both upper and lower gastrointestinal endoscopy has been established, requiring highly technical skills with a well-documented learning curve. These procedures require advanced training and a highly skilled practitioner, so hence remain largely provider-dependent. Given the variation in skills between providers, 11.3% of upper gastrointestinal neoplasms are missed on the initial upper endoscopy, and 2.1–5.9% of colorectal polyps or cancers are missed on colonoscopy [9]. Research has shown that each 1% increase in adenoma detection rate could translate to a 3% decrease in the risk of colorectal cancer [10]. Advancements in endoscopy are focused on two main areas. Firstly, to improve polyp detection and reduce polyp miss rates, especially for sessile polyps. Secondly, once polyps have been detected, there is a need to minimise the resection of hyperplastic polyps following the ‘diagnose and leave’ strategy [11]. To achieve this, there is a need to develop techniques for in situ classification of detected polyps.

Colonoscopy remains the gold standard investigation of the lower gastrointestinal tract, with pattern recognition of colorectal lesions such as polyps and cancer dependent on the endoscopist. Recent developments in AI technology have focussed on detailed real-time analysis of colonoscopic images and videos of diminutive polyps for identification and characterisation. However, the majority of developments remain in the training and validation phases.

Current data suggests that 26% of diminutive pre-malignant lesions and 3.5% of colorectal cancers are missed during colonoscopy, even with advancing imaging techniques, such as narrow-band imaging (NBI) [12,13,14]. The NBI International Colorectal Endoscopic Classification or the Japan NBI Expert Team classifications allow endoscopists to make histological predictions of diminutive polyps by detailed pit pattern recognition and microvasculature. Nevertheless, clinician diagnosis is subjective, relying on endoscopists’ experience. Comparative analyses have shown AI to be beneficial to novice endoscopists with lower diagnostic capabilities of clinically indeterminate lesions and to reduce inter-observer variability [1,3]. The current literature in upper gastrointestinal endoscopy similarly demonstrates a potential but limited application for use in its current state with endoscopist diagnosis superior to the AI outputs [15].

The role of AI in endoscopy needs to be further defined as an adjunct to facilitate real-time classification and discrimination of lesions with increased accuracy and efficiency. The aim of this study is to explore the role and efficacy of artificial intelligence in real-time histological prediction of colorectal lesions during colonoscopy.

## 2. Methods

### 2.1. Search Strategy

A systematic search was performed using EMBASE (OvidSP) and MEDLINE (OvidSP) to identify potentially relevant articles published between 1966 and 6 August 2023 using the Preferred Reporting Items for Systematic Reviews and Meta-Analyses (PRISMA) guidelines [16]. A systematic search strategy comprising keywords and MeSH headings relating to “artificial intelligence”, “machine learning”, “computer-aided”, “colonoscopy”, and “colon/rectum/colorectal” used in combination with Boolean operators AND and OR was conducted. Only completed studies were considered for inclusion. The search criteria detailing the combination of terms used are shown in Supplementary Table S1.

### 2.2. Eligibility Assessment and Data Extraction

Studies reporting on prospectively designed, real-time prediction of histology of colorectal lesions during colonoscopy were selected for further review. Specific inclusion criteria were real-time artificial assessments and histological predictions. All types of AI systems were included. Studies were excluded if they primarily used images or videos retrospectively and/or from datasets. Review articles, case reports, editorials, opinions, conference abstracts, news articles, and articles not written in the English language were excluded. Two independent reviewers (BV, MT) screened all titles and abstracts to identify articles meeting the criteria for full-text review. Reference lists of selected articles were screened to identify additional relevant articles.

Parameters for data extraction from the full-text review included the number of patients and polyps detected, the type of AI platform used and their previous validation methods, and primary outcomes and results, including sensitivity, specificity, positive predictive value, negative predictive value, and area under the curve (AUC).

### 2.3. Outcomes

The primary outcome was to identify the diagnostic accuracy of various AI platforms in the histological prediction of colorectal polyps. Single-arm studies and those with a comparative group (for example, neoplastic versus non-neoplastic lesions and CAD platforms versus endoscopists) were included.

### 2.4. Statistical Analysis

Descriptive information, including the country of the study, the number of patients enrolled, the number of lesions detected, and the type of AI platform used, was collected. Forest plots were created to demonstrate the diagnostic performance of CAD (machine intelligence) and endoscopists (human intelligence) using the sensitivity of histological prediction of the lesions. An overall pooled estimate of sensitivity and specificity, with their reported 95% confidence interval, was performed to assess CAD performance between selected articles (RevMan Version 5.4).

## 3. Results

**Figure 1:**
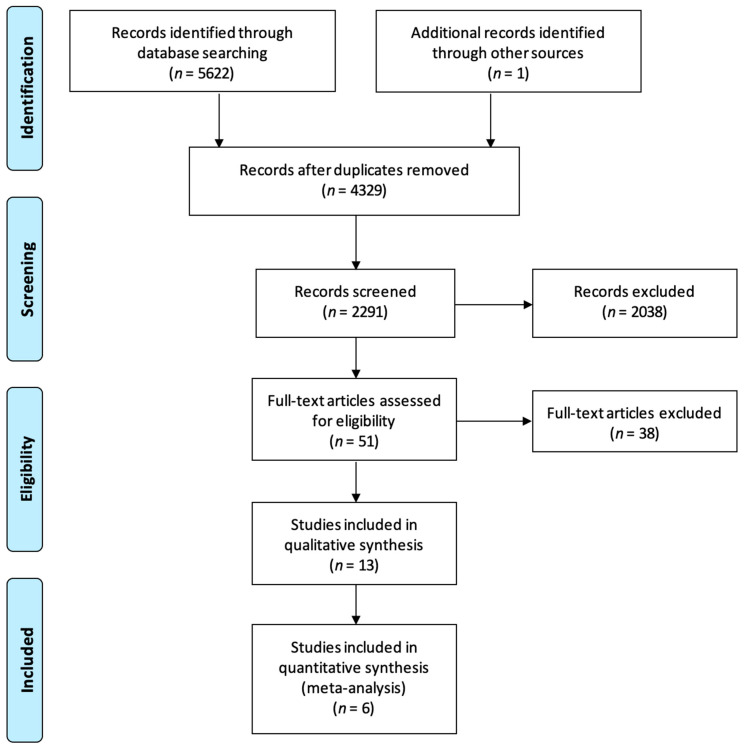
PRISMA flowchart demonstrating article selection.

A total of 2290 articles were screened using the titles and abstracts. A full-text review was performed on 51 articles, and a final 13 articles were included for analysis. Detailed article selection is described in Figure 1.

A total of 2958 patients and 5908 colorectal lesions were included in the analysis. Six studies used Japanese populations, followed by one study performed in Singapore (patients, *n* = 1514; lesions, *n* = 2876) [17,18,19,20,21,22,23]. Studies from the Western world included three from the USA and one each from the UK, The Netherlands, Norway, Canada, and Brazil (patients, *n* = 1444, lesions, *n* = 3032) [19,24,25,26,27,28,29]. There was an equal distribution of studies from the Eastern and Western world.

**Figure 2:**
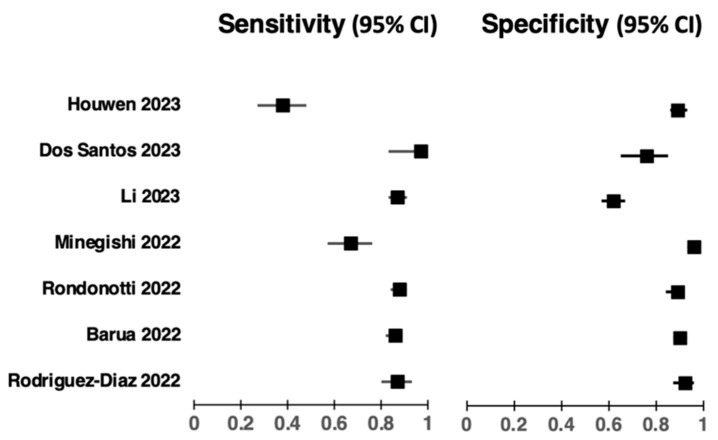
Forest plots demonstrating the sensitivity (95%) and specificity (95%) across seven studies assessing the diagnostic performance of AI platforms in colonoscopic real-time histological prediction of colorectal lesions [18,19,22,24,25,26,27].

Nine studies used an Olympus colonoscopy module, three studies used Fujifilm, and one study did not specify. Ten studies assessed colorectal lesions, and three studies assessed rectosigmoid lesions specifically. All studies aimed to distinguish neoplastic lesions from benign lesions, and two studies in particular assessed diminutive lesions. The study characteristics are summarised in Table 1, and diagnostic performance is detailed in Table 2 and Figure 2.

**Figure 3:**
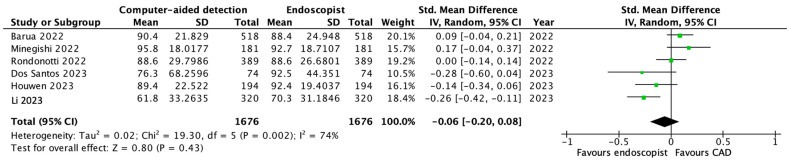
Forest plot demonstrating no significant difference in distinguishing colorectal neoplastic and non-neoplastic lesions when comparing endoscopist diagnosis to CAD output (*p* = 0.43) [18,19,22,25,26,27]. Green square = sensitivity with 95% CI.

Six studies assessed a CAD-AI system with direct comparison to the standard endoscopist interpretation of a lesion. Analysis using the sensitivity of each test (endoscopist versus CAD) revealed that endoscopist diagnosis was favourable to a CAD platform, although no statistical significance was reached (*p* = 0.43)—Figure 3.

### 3.1. Elastic Scattering Microscopy

Elastic scattering microscopy (ESS) uses short light pulses of 50 microseconds covering a wavelength of 300–900 nm (ultraviolet to infrared spectra) [24,30]. The short pulses reduce the influence of the surrounding lighting for higher-quality detection of lesions. The ESS comprises optical probes with two columns of fibres (200 μm) for illumination and detection. The probes allow the assessment of a tissue depth of 350 μm and a tissue volume of less than 0.2 mm^3^. The probes can be built into the biopsy forceps to appear between the jaws or affixed adjacent to the biopsy forceps. The integrated ESS probe has direct contact with the lesion for spectroscopic optical biopsies and a binary output of neoplastic and non-neoplastic. To account for the spectral light variations, calibration is performed with a white colour. Rodriguez-Diaz et al. demonstrated a sensitivity of 0.92, specificity of 0.87, and NPV of 0.87 for distinguishing neoplastic polyps [24]. Diminutive polyps achieved a sensitivity of 0.91, specificity of 0.88, and NPV of 0.89 [24]. Overall, ESS demonstrated high sensitivity for characterising polyps.

### 3.2. Autofluorescence Imaging

Autofluorescence imaging (AFI) utilises real-time analysis of colour ratios of red (R), blue (B), and green (G). Each colour is represented by an integer standardised by the International Electrotechnical Commission and accounts for the intensity of light emitted to a charge-coupled device [20]. The output is assessed on an endoscopic monitor for real-time analysis. The principle is based on the G/R ratio correlating with the intensity of the lesion distinguishing neoplasia. Aihara et al. demonstrated a cut-off value of 1.01, demonstrating that a ratio less than 1.01 was suggestive of neoplasia, and a ratio greater than 1.01 was non-neoplastic, yielding a sensitivity of 0.94, specificity of 0.89, and NPV 0.85 [20]. Inomata explored real-time AFI in 2013 by identifying potential lesions initially with NBI and/or chromoendoscopy. A G/R cut-off value of 0.89 was discriminatory, with a ratio less than 0.89 indicative of neoplasia [23]. Furthermore, a ratio of 0.77 was suggestive of submucosal deep cancers [23].

### 3.3. Narrow Band Imaging, Magnification Analysis, Supper Vector Machine

Narrow band imaging (NBI) has made marked advances in the characterisation of lesions during colonoscopy by endoscopists’ assessment of the microvasculature and pit-pattern recognition using a filtered xenon light (for shorter wavelength). The system was taught to recognise target features and categorise lesions into three types, and the SVM output differentiated neoplastic (>0.5) and non-neoplastic lesions (≤0.5) automatically [17]. Kominami et al. demonstrated a sensitivity of 0.93, specificity of 0.93, and NPV of 0.93 with this novel system with an SVM output [17]. Barua et al. trialled a similar system named EndoBRAIN with an analysis of 892 polyps showing a sensitivity of 0.90 and specificity of 0.86 with CAD with SVM compared to visual inspection (sensitivity 0.88, specificity 0.83) [19].

Studies showed an increase in neoplastic lesion detection with CAD; however, these were not statistically significant. Mori et al. used NBI followed by methylene blue staining (for visualising cellular architecture) with a three-step algorithm: (1) feature analysis, (2) lesion classification with SVM, and (3) histopathology prediction [21]. The NBI and staining method of 466 diminutive polyps predicted histopathology 98.1% of the time with an NPV of 0.96 [21]. Houwen et al. created the polyp artificial recognition system (POLAR), which characterised features from polyps in NBI mode with a 0.89 sensitivity compared to 0.92 with endoscopists without a significant difference [27].

### 3.4. White Light Imaging and Narrow Band Imaging

White light imaging (WLI) is based on the diffuse reflectance of a xenon white light where multiple wavelengths of white light are scattered and absorbed in the tissues. Shahidi et al. used WLI and NBI in a deep convolutional neural network setting to demonstrate a 71.1% concordance with histopathological diagnosis of diminutive lesions [29]. Although the clinical decision support solution (CDSS) agreed with endoscopic diagnosis 89.6% of the time, there remains a discrepancy with histology results [29]. Minegishi et al.’s approach used WLI to confirm colorectal lesions, after which NBI was employed, and an in-built CAD software was used to characterise polyp detection [22]. With NBI-CAD, the overall sensitivity for detection was increased from 0.93 to 0.96, although it was not statistically significant [22].

### 3.5. Blue Light Imaging (CAD-EYE System)

Blue light imaging (BLI) has been utilised in a real-time convolutional neural network AI system. BLI is based on two monochromatic lasers at 410 nm and 450 nm wavelengths to assess microvasculature patterns. The CAD-EYE system based on pattern recognition showed an optical diagnosis in 92.3% of cases, with poorer performance in non-experts (82.3%) compared to experts (91.9%) [26]. However, for non-expert endoscopists, CAD-EYE improved their diagnostic performance from 81.8% to 86.2% [26]. Li et al. also used CAD-EYE, demonstrating a higher sensitivity for endoscopist diagnosis (sensitivity 0.70) compared with CAD-EYE (sensitivity 0.62) [18]. Dos Santos et al. utilised BLI in addition to WLI with magnification to show a sensitivity of 0.76 compared with endoscopist analysis at 0.94 [25]. All convolutional neural networks were shown to be inferior to the standard endoscopist diagnosis.

### 3.6. Other

Quan et al. used the EndoVigilant platform, which was established in the USA based on features extracted from real-time colorectal lesion detection to provide an output to aid in the diagnosis [28]. The EndoVigilant system displayed a tendency towards improved identification compared to historical standard diagnoses; however, no statistical significance was achieved [28].

A summary of the AI technologies is detailed in Table 3.

## 4. Discussion

Colorectal cancer remains the third most common cancer globally, with more than 1.9 million new cases annually [31]. Histopathological analysis remains the gold standard for definitive diagnosis; however, clinical expertise in pattern recognition of polyps is vital for early polyp cancer diagnosis. This study reviewed the diagnostic performance of real-time CAD systems in predicting histopathology of colorectal lesions. Thirteen studies included in the analysis used six different platforms with various adaptations of them. The systems were trained using still images of polyps/lesions, feature identification, extraction, and classification into the defined groups (neoplastic versus non-neoplastic). A meta-analysis of six of the thirteen studies comparing endoscopist and CAD diagnosis demonstrated superiority with the current standard of endoscopist diagnosis. Rondonotti et al. demonstrated that CAD-assisted diagnosis was beneficial for junior endoscopists; however, this waned with an increase in expertise. AI applications have shown encouraging results in optical biopsies using NBI, WLI, and BLI with associated magnification. Other techniques, such as autofluorescence imaging using colour ratios and elastic scattering microscopy, are less common but show equal promise. The current literature shows comparable advances in real-time histological analysis in the Eastern and Western worlds, demonstrating the external validity of the presented results.

Advancements in light technologies such as narrow band imaging and white light imaging with magnification have allowed improved and more detailed analysis of the microvasculature and pit patterns. Naturally, junior endoscopists’ skills and interpretation require nurturing to identify the subtleties associated with high-risk lesions. However, at any level, artificial intelligence-based platforms can provide an important adjunct for the optical diagnosis of polyps, which may benefit from excision. Furthermore, the implementation of AI-based platforms may decrease the heterogeneity between endoscopists. Overall, CAD-assisted platforms are expected to improve optical diagnosis, decision for polypectomy, and time efficiency.

Although much research is underway in establishing the value of AI platforms in gastrointestinal endoscopy, it has had more success in radiology. McKinney et al. demonstrated that the AI system outperformed radiologists in breast cancer detection in mammography across large UK and USA datasets [32]. Similarly, a Swedish group showed a double screening of mammograms with AI, resulting in a 4% higher non-inferior cancer detection rate [33]. The national breast screening programme has published a report to further the use of AI in breast cancer screening, highlighting the real-world value of AI in healthcare. Industrial partners such as Google’s DeepMind Health are contributing to building robust platforms with deep neural networking to mimic the human mind for early diagnosis decision-making and provide training digitally. Furthermore, CAD platforms have been associated with significant cost-savings, demonstrating a 4.8% incremental gain in colorectal cancer incidence in the screening tools facilitated with AI [34].

AI in radiology has advanced far more rapidly than endoscopy, not only in the diagnostic setting but for metastatic surveillance and prognostication. However, AI use in endoscopy requires more focussed development. Still, image recognition has achieved better outcomes than real-time dynamic images. Earlier adenoma detection is a fundamental need to prevent the progression of cancer. Polyp recognition and characterisation is an endoscopist skill; however, subjective. An algorithm based on feature analysis, including microvascular pit pattern during colonoscopy, can facilitate a decision to excise a potential adenoma, particularly with junior endoscopists. This technology will facilitate the decision for polyp excision and reduce the need for a repeat colonoscopy.

There is a noticeable paradigm shift in medical diagnostics with the application of AI platforms to improve the timely interpretation of interventions to direct further management. Prior to the implementation of new technology within healthcare systems, it is important to critique the current literature and ensure robust studies for safe translation, implementation, and maintaining patient safety. Most evidence supporting diagnostic algorithms has been published without AI-specific reporting guidelines. The STARD-AI Steering Group is developing an AI-specific extension to the STARD statement to complement the EQUATOR (Enhancing Quality and Transparency of Health Research) network program, CONSORT-AI (Consolidated Standards of Reporting Trials), SPIRIT-AI (Standard Protocol Items: Recommendations for Interventional Trials) and TRIPOD-ML (Transparent Reporting of a Multivariable Prediction Model for Individual Prognosis or Diagnosis) [35,36,37].

However, it is important to consider the ‘black box’ associated with AI systems, where internal biases are not apparent or assessable by the users. A sophisticated deep learning algorithm utilises specific data points and features correlation to produce a clinically relevant output. However, there is no rationalisation of the decision-making process and the in-built mechanisms, making this a complex system to understand for clinicians and developers. In the real world, there would be concerns for clinical auditing.

The implementation of AI in healthcare faces several obstacles that will need to be addressed. These obstacles arise at all levels of AI adoption, including data collection concerns, algorithm development concerns, ethical and societal concerns, and clinical implementation concerns with the lack of empirical data validating the effectiveness of AI-based platforms in clinical trials [38].

### Limitations

There is much-published literature on AI use in colonoscopy using still images and retrospective videos. However, the true test for its clinical utility comes from prospective data. A number of limitations still exist. The complexity of colorectal disease means the input features need to be refined to prevent causing false classifications. For example, background inflammation may impact feature extraction. It is important to mitigate the number of false negatives, and therefore, AI is routinely applied on withdrawal only. Colorectal AI algorithms benefit from larger datasets; however, data thus far show its inferiority to experienced endoscopists. It is important to note the heterogeneity of the AI applications, with variable iterations of the same platform. Data so far shows AI use in combination with expert opinion, not alone. Pooled analyses were not performed due to variability in the platforms, diagnostic parameters, and cut-off values. Due to the heterogeneity in the AI platforms used, it was not possible to perform a hierarchical summary of receiver operating curves or bivariate analysis. More robust studies on the same platform would generate more accurate and reliable data for users and policymakers. Currently, comparative studies are limited in interpreting clinical utility. More focus on developing a real-time system that is superior to current practice is required. Once these systems are tested and validated, randomised controlled trials comparing these platforms with each other and with current clinical practice will be required prior to clinical translation.

## 5. Conclusions

AI applications have shown encouraging results in distinguishing neoplastic and non-neoplastic colorectal lesions. Current endoscopic advancements with NBI have achieved significantly improved real-time histological predictions. However, AI research largely focuses on still image analysis, with few prospective studies that did not demonstrate a significant improvement with the addition of AI. Although there was a diagnostic improvement with junior endoscopists, it failed to equal that of expert endoscopists. With expert endoscopists’ diagnoses superior to current prospective models, there is further work needed to improve the characterisation of the lesions before clinical translation. However, there is potential in the proposed use of AI in colonoscopy for improved diagnostic capabilities and time efficiency.

## Figures and Tables

**Table 1:** Study characteristics of 13 articles selected for review. NR = not reported.

Year	Author	Country	Number of Patients	Number of Lesions Analysed	Site	Colonoscopy Module Used	Type of System	How Were the Systems Validated
2012	Aihara et al. [20]	Japan	32	102	Colorectal	Olympus Corp	Autofluorescence endoscopy	NR
2013	Inomata et al. [23]	Japan	88	163	Colorectal	CF-FH260AZI, Olympus	Autofluorescence endoscopy	NR
2016	Kominami et al. [17]	Japan	48	118	Colorectal	Olympus	NBI, magnifying colonoscopy with a support vector machine	Training set: 2247 images from 1262 colorectal lesions
2018	Mori et al. [21]	Japan	327	475	Rectosigmoid	CF-Y-0058 Olympus	Endocytoscope with light microscopy NBI mode and methylene blue staining	Training: 61,925 images
2020	Shahidi et al. [29]	Canada	-	644	Colorectal	Olympus	White light and NBI	Previously trained and validated
2021	Rodriguez-Diaz et al. [24]	USA	169	367	Colorectal	NR	Elastic-scattering spectroscopy	Training set: 512 measurements from 294 polyps
2022	Barua et al. [19]	Norway/ UK/Japan	518	892	Rectosigmoid	Olympus Corp	High-resolution magnification colonoscopies, NBI, SVM	Previous training and validation: 35,000 polyps images from five Japanese endoscopy centres
2022	Rondonotti et al. [26]	USA	389	596	Rectosigmoid	ELUXEO 7000 endoscopy, Fujifilm	Blue light imaging	Previously validated
2022	Quan et al. [28]	USA	100	-	Colorectal	CF-HQ190 Olympus	Endovigilant	Training: 83,000 images from 300 colonoscopy videos. Validation: 21,454 images from 30 videos—sensitivity 0.90, specificity 0.97, AUC 0.94
2022	Minegishi et al. [22]	Japan	181	465	Colorectal	EVIS-X1 Olympus	White light and NBI	Training: 18,079 images
2023	Li et al. [18]	Singapore	320	661	Colorectal	ELUXEO 7000 endoscopy, Fujifilm	CNN with blue laser imaging	Commercially available tool
2023	Dos Santos et al. [25]	Brazil	74	110	Colorectal	Fujifilm	Magnification with multi-light technology (WLI and link colour imaging)	NR
2023	Houwen et al. [27]	The Netherlands	194	423	Colorectal	Olympus	POLyp Artificial Recognition	Training: Eight hospitals collected 2637 annotated images from 1339 polyps

**Table 2:** Diagnostic performance of AI-assisted colonoscopy. CI = confidence interval 0.95, PPV = positive predictive value, NPV = negative predictive value, *p* < 0.05 is considered statistically significant, NR = not reported.

		Computer Assisted Diagnosis					Endoscopist Diagnosis					
Year	Author	Sensitivity (CI)	Specificity (CI)	PPV (CI)	NPV (CI)	Accuracy (CI)	Sensitivity (CI)	Specificity (CI)	PPV (CI)	NPV (CI)	Accuracy (CI)	*p* Value
2012	Aihara et al. [20]	0.94	0.89	0.96	0.85	NR	NR	NR	NR	NR	NR	NR
2013	Inomata et al. [23]	0.84	0.83	0.53	0.96	0.83	NR	NR	NR	NR	NR	NR
2016	Kominami et al. [17]	0.96	0.93	0.96	0.93	0.95	NR	NR	NR	NR	NR	NR
2018	Mori et al. [21]	NR	NR	NR	0.96 (0.92–0.99)	NR	NR	NR	NR	0.92 (0.88–0.95)	NR	NR
2020	Shahidi et al. [29]	NR	NR	NR		NR	NR	NR	NR	NR	NR	NR
2021	Rodriguez-Diaz et al. [24]	0.92 (0.87–0.96)	0.87 (0.80–0.93)		0.87 (0.80–0.93)	0.91	NR	NR	NR	NR	NR	NR
2022	Barua et al. [19]	0.90 (0.87–0.93)	0.86 (0.82–0.89)	0.82 (0.78–0.86)	0.93 (0.90–0.95)		0.88 (0.84–0.92)	0.83 (0.79–0.86)	0.79 (0.74–0.83)	0.92 (0.89–0.94)	NR	NR
2022	Rondonotti et al. [26]	0.89 (0.84–0.91)	0.88 (0.84–0.91)	0.85 (0.80–0.89)	0.91 (0.87–0.94)	0.92 (0.85–0.91)	0.89 (0.84–0.92)	0.89 (0.85–0.92)	0.86 (0.81–0.90)	0.91 (0.87–0.94)	0.89 (0.86–0.91)	NR
2022	Quan et al. [28]	NR	NR	NR	NR	NR	NR	NR	NR	NR	NR	NR
2022	Minegishi et al. [22]	0.96 (0.93–0.98)	0.67 (0.57–0.76)	0.89 (0.84–0.92)	0.86 (0.76–0.93)	0.88 (0.84–0.91)	0.94 (0.90–0.95)	0.63	NR	0.86	NR	NR
2023	Li et al. [18]	0.62 (0.57–0.67)	0.87 (0.83–0.91)	0.89 (0.85–0.92)	0.59 (0.54–0.64)	0.72 (0.68–0.75)	0.70 (0.66–0.75)	0.83 (0.78–0.87)	0.87 (0.83–0.90)	0.63 (0.58–0.69)	0.75 (0.72–0.78)	0.001
2023	Dos Santos et al. [25]	0.76 (0.65–0.85)	0.97 (0.83–1.00)	0.98 (0.91–1.00)	0.60 (0.45–0.74)	0.82 (0.79–0.85)	0.93 (0.84–0.97)	0.97 (0.83–1.00)	0.99 (0.93–1.00)	0.83 (0.66–0.93)	0.94 (0.92–0.95)	<0.01
2023	Houwen et al. [27]	0.89 (0.86–0.93)	0.38 (0.27–0.48)	0.86 (0.82–0.89)	0.46 (0.34–0.58)	0.79 (0.75–0.83)	0.92 (0.90–0.95)	0.44 (0.33–0.55)	0.87 (0.84–0.91)	0.58 (0.46–0.70)	0.83 (0.79–0.86)	0.1

**Table 3:** Summary of the current artificial intelligence platforms used for real-time histological detection of colorectal lesions.

Artificial Intelligence System	Technology	System Integration	Detection
Electric scattering microscopy	Short light pulses of 50 microseconds encompassing wavelengths of 300–900 nm.	Optical probes with two 200 μm columns of fibres for illumination and lesion detection. Probes can be built into biopsy forceps.	Spectroscopic optical biopsy with binary output: neoplastic vs. non-neoplastic.
Autofluorescence imaging	Real-time analysis of colour ratios of red, blue, and green. The green/red ratio represents the intensity of light on the lesion.	The intensity of light is emitted to a charge-coupled device and displayed on the endoscopic monitor.	A cut-off value of the green/red ratio was distinguishable between neoplastic and non-neoplastic lesions.
Narrow band imaging, magnification, and support vector machine	Algorithm recognising target features, including microvasculature and pi-patterns, using a filtered xenon light (shorter wavelength).	Support vector system outputs from targeted feature analysis using narrow-band imaging.	Lesion characterisation using a cut-off value to differentiate neoplastic and non-neoplastic lesions.
White light imaging and Narrow band imaging	Diffuse reflectance of a xenon light where multiple wavelengths are absorbed in tissues.	Algorithm incorporating features from white light and narrow band imaging for characterisation (deep convolutional neural network).	Lesion characterisation using feature analysis.
Blue light imaging (CAD-EYE system)	Blue light imaging is based on two monochromatic lasers at 410 nm and 450 nm wavelength to assess microvasculature patterns.	Real-time convolutional neural network system based on pattern recognition.	Optical diagnosis distinguishing neoplastic and non-neoplastic lesions.
EndoVigilant	Video and augmentation of lesion attributes.	Real-time computer-aided outputs on the endoscopic screen.	Lesion attributes are displayed on the endoscopic screen to aid in diagnosis.

## Supplementary Materials

The following supporting information can be downloaded at: https://www.mdpi.com/article/10.3390/diagnostics13203267/s1, Table S1: Detailed search strategy for article identification.
