# Supplementary material for: The Role of Artificial Intelligence in Prospective Real-Time Histological Prediction of Colorectal Lesions during Colonoscopy: A Systematic Review and Meta-Analysis
Source: Diagnostics (Basel). 2023 Oct 20;13(20):3267. doi: 10.3390/diagnostics13203267 (PMC10606449; doi:10.3390/diagnostics13203267)
Supplement: Supplementary file 1 [file diagnostics-13-03267-s001.zip › diagnostics-2671706-supplementary.pdf]

Supplementary table S1: Search strategy to identify eligible articles for the systematic review.

Database	Searches	Results
EMBASE	artificial intelligence.mp.	104079
	AI.mp.	177311
	machine learning.mp.	193600
	deep machine.mp.	561
	exp artificial intelligence/	83362
	(computer-aided or computer aided).mp.	77792
	1or 2 or 3 or 4 or 5 or 6	500201
	(colorectal or colon or rectum).mp.	1490474
	exp colon/	112270
	8 or 9	1496654
	colonoscopy.mp.	207143
	exp colonoscopy/	103984
	11 or 12	208146
	7 and 10 and 13	3155
	remove duplicates from 14	**2440**
OVID	artificial intelligence.mp.	94748
	AI.mp.	158997
	machine learning.mp.	157780
	deep machine.mp.	492
	exp artificial intelligence/	176492
	(computer-aided or computer aided).mp.	67377
	1or 2 or 3 or 4 or 5 or 6	510381
	(colorectal or colon or rectum).mp.	1076512
	exp colon/	74988
	8 or 9	1076512
	colonoscopy.mp.	144089
	exp colonoscopy/	35024
	11 or 12	147439
	7 and 10 and 13	2467
	remove duplicates from 14	**1888**
